# Anomalous systemic arterial supply to the basal segment of the left lung with large vessel giant cell arteritis demonstrated by 18F-FDG PET/CT

**DOI:** 10.1007/s12055-026-02252-2

**Published:** 2026-08-05

**Authors:** Takanori Ichikawa, Megumi Kubota, Dai Kishida, Yasuhiro Shimojima, Yoshiki Sekijima

**Affiliations:** 1 Department of Medicine (Neurology and Rheumatology), Shinshu University School of Medicine, Matsumoto, 3-1-1, Asahi, Matsumoto-shi, Nagano, 390-8621 Japan; 2 Department of Neurology, Aizawa Hospital, Matsumoto, Nagano, Japan

**Keywords:** Anomalous systemic arterial supply to the basal segment of the left lung, Large-vessel giant cell arteritis, Aortitis, Medical and surgical management

## Abstract

We report a rare case of anomalous systemic arterial supply to the basal segment of the left lung (ABLL) coexisting with large-vessel giant cell arteritis (LV-GCA) in a 73-year-old woman. 18F-fluorodeoxyglucose (FDG) positron emission tomography/computed tomography (PET/CT) demonstrated inflammatory involvement of both the aorta and the anomalous artery. To reduce vascular and perioperative risks, immunosuppressive therapy was initiated first, resulting in improvement in clinical and radiological findings. This case highlights the importance of controlling active vasculitis before definitive surgical treatment.

A 73-year-old woman presented with a persistent dry cough and weight loss. Chest radiography and computed tomography (CT) revealed a mass-like lesion in the basal segment (S10) of the left lower lobe, initially raising suspicion of lung malignancy (Fig. 1A). 18F-fluorodeoxyglucose (FDG) positron emission tomography/computed tomography (PET/CT) revealed diffuse FDG uptake along the anomalous systemic artery supplying the left lower lobe maximum standardized uptake value (SUVmax) of 4.2 as well as the aortic wall and its major branches including the carotid, subclavian, iliac, and femoral arteries (Fig. 1B). Laboratory investigations showed elevated inflammatory markers, including C-reactive protein (4.58 mg/dL) and erythrocyte sedimentation rate (107 mm/h). Autoimmune serology and infectious disease screening were negative. Contrast-enhanced CT demonstrated diffuse circumferential thickening of the aortic wall and major arterial walls and identified an anomalous systemic artery arising from the descending aorta and supplying the left lower lobe without evidence of lung sequestration (Fig. 1C). Given the normal bronchial anatomy, the patient was diagnosed with anomalous systemic arterial supply to the basal segment of the left lung (ABLL) and, according to the 2022 American College of Rheumatology/European League Against Rheumatism (ACR/EULAR) classification criteria, large-vessel giant cell arteritis (LV-GCA) [1].

**Fig. 1:**
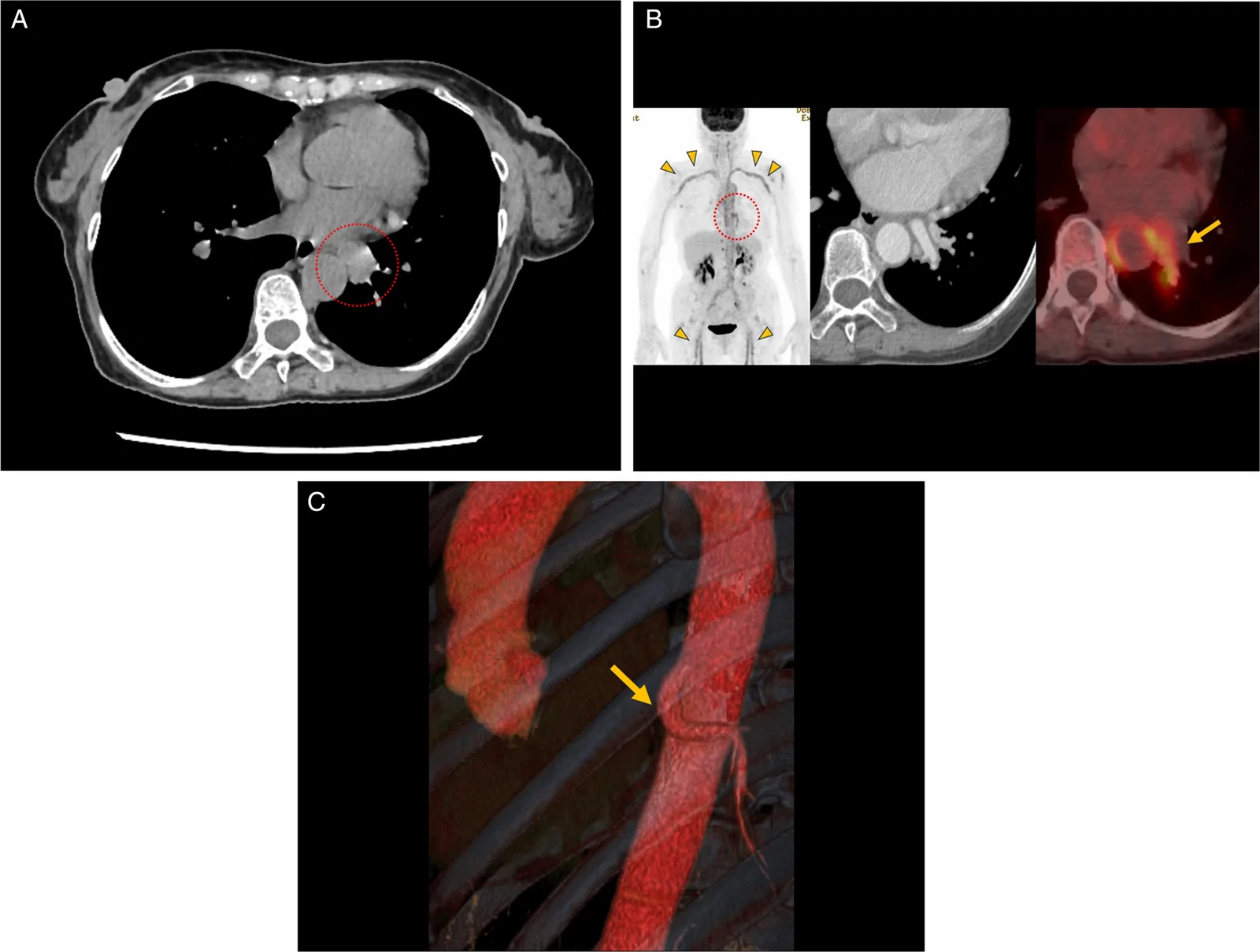
**A** Plain chest computed tomography reveals a mass-like lesion in the lower left lung field (dotted circle). **B**. 18F-fluorodeoxyglucose (FDG) positron emission tomography/computed tomography (PET/CT) showing FDG uptake in the major branches of the aorta (arrowhead) and S10 region of the left lung (dotted circle); this area corresponds to an anomalous artery branching from the descending aorta, which showed increased FDG uptake (arrow). **C**. Contrast-enhanced computed tomography demonstrates an anomalous vessel arising from the descending aorta (arrow)

ABLL is a rare congenital vascular anomaly characterized by systemic arterial supply to normal lung parenchyma and is distinct from classical pulmonary sequestration [2]. Chronic high-flow hemodynamics predispose affected vessels to complications such as hemoptysis, pulmonary hypertension, and aneurysm formation. In most cases, surgeons perform treatment involving ligation or division of the anomalous artery combined with lobectomy [3]. In the present case, active LV-GCA further increased the risk of vascular complications through combined mechanical stress and inflammatory injury. After discussion with the thoracic surgery team, surgical treatment was deferred until adequate remission of vasculitis was achieved, in accordance with EULAR recommendations to minimize perioperative vascular risks [4].

Prednisolone (40 mg/d), followed by azathioprine (50 mg/d), resulted in rapid normalization of inflammatory markers and marked improvement in wall thickening of the large vessels and anomalous artery (Fig. 2). The cough and weight loss gradually resolved, likely reflecting reduced vasculitis-related airway irritation and systemic inflammation. The patient reduced the prednisolone dosage without relapse during 7 months of follow-up. The thoracic surgery team continues close follow-up until surgery can be performed after adequate control of aortitis with low-dose prednisolone.

**Fig. 2:**
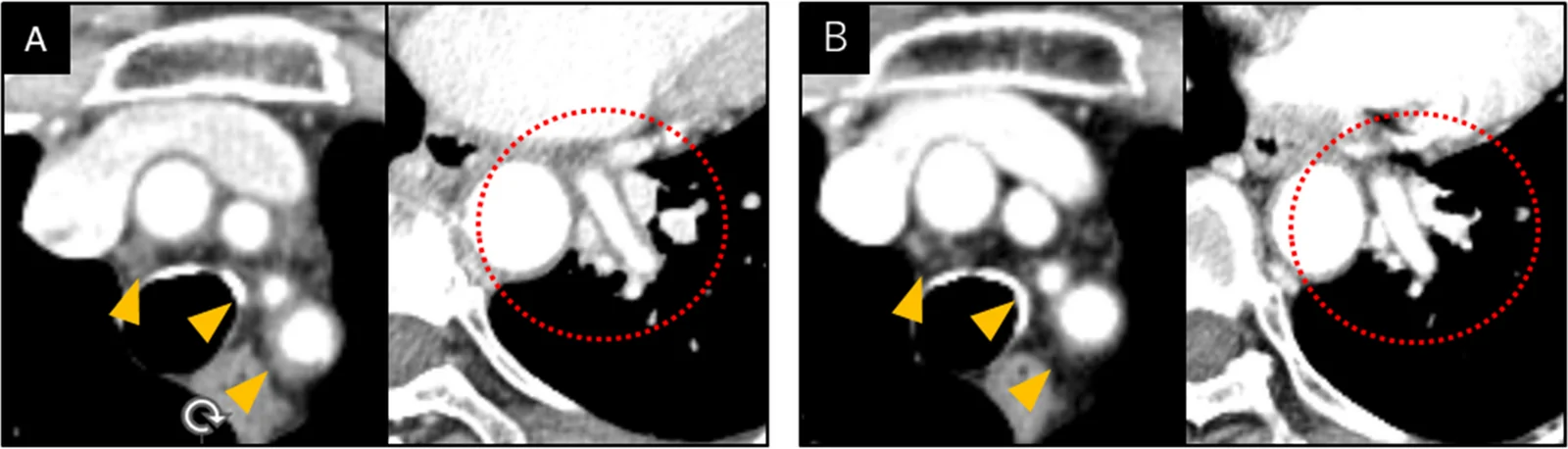
Combined treatment with prednisolone and azathioprine resulted in improvement in arterial wall thickening (arrowhead and dotted circle). (**A**) Before treatment. (**B**) Five months after treatment

The coexistence of ABLL and active LV-GCA increases vascular risks through combined hemodynamic and inflammatory stress, highlighting the importance of controlling inflammation before surgery. In such cases, close collaboration between surgical and medical teams may be essential to ensure that surgery is performed at the optimal time.

## Data Availability

Not applicable as no datasets were generated or analyzed.

## Declarations

### Artificial intelligence (AI) declaration

The authors declare that no generative AI or AI-assisted technologies were used in the preparation of this manuscript.
